# Changing the Paradigm for the Treatment and Development of New Therapies for FSGS

**DOI:** 10.3389/fped.2016.00025

**Published:** 2016-03-23

**Authors:** Cathie Spino, Jordan S. Jahnke, David T. Selewski, Susan Massengill, Jonathan Troost, Debbie S. Gipson

**Affiliations:** ^1^Department of Biostatistics, School of Public Health, University of Michigan, Ann Arbor, MI, USA; ^2^NephCure Accelerating Cures Institute, King of Prussia, PA, USA; ^3^Department of General Internal Medicine, University of Pennsylvania, Philadelphia, PA, USA; ^4^Department of Pediatrics, School of Medicine, University of Michigan, Ann Arbor, MI, USA; ^5^Department of Pediatrics, Division of Nephrology, Carolinas Medical Center, Charlotte, NC, USA

**Keywords:** FSGS, nephrotic syndrome, therapy, adverse effects, clinical trials

## Abstract

Focal segmental glomerulosclerosis (FSGS) is a renal pathology finding that represents a constellation of rare kidney diseases, which manifest as proteinuria, edema nephrotic syndrome, hypertension, and increased risk for kidney failure. Therapeutic options for FSGS are reviewed displaying the expected efficacy from 25 to 69% depending on specific therapy, patient characteristics, cost, and common side effects. This variability in treatment response is likely caused, in part, by the heterogeneity in the etiology and active molecular mechanisms of FSGS. Clinical trials in FSGS have been scant in number and slow to recruit, which may stem, in part, from reliance on classic clinical trial design paradigms. Traditional clinical trial designs based on the “learn and confirm” paradigm may not be appropriate for rare diseases, such as FSGS. Future drug development and testing will require novel approaches to trial designs that have the capacity to enrich study populations and adapt the trial in a planned way to gain efficiencies in trial completion timelines. A clinical trial simulation is provided that compares a classical and more modern design to determine the maximum tolerated dose in FSGS.

## Introduction

Focal segmental glomerulosclerosis (FSGS) manifests with proteinuria, hypertension, and in the worse cases progresses to kidney failure. FSGS is a renal pathology finding that represents a constellation of rare kidney diseases and results in a significant public health burden accounting for 5% of adults and 12% of children with incident end stage kidney disease (ESKD) in the US annually (1). Broadly, FSGS describes a kidney scarring pattern that occurs in a focal and segmental pattern, but does not describe the underlying pathophysiology. FSGS is often classified as primary or secondary (e.g., following systemic illnesses IgA nephropathy, post-infectious glomerulonephritis). Genetic causes of FSGS may represent a distinct type that does not fit well into this classification. In general, the distinction between primary, secondary, and genetic causes of FSGS have classically driven therapeutic decision-making and clinical trial design. However, emerging precise molecular mechanisms may represent distinct endophenotypes of FSGS, which may also vary temporally by disease initiation, maintenance, or progression.

Despite the significant patient and health burden, there is a paucity of therapeutic options for those with FSGS. Therapies available include immunosuppression, renin–angiotensin–aldosterone blockade, lipid lowering agents, and other blood pressure lowering agents as necessary. Unfortunately, the available immunosuppression therapies have a significant toxicity profile that may be dose limiting. Side effects, such as those altering physical appearance (e.g., alopecia, hirsutism, and weight gain) or physical function (e.g., weakness, tremor, and infertility), may contribute to poor adherence. These decisions are further complicated by those with monogenetic forms of FSGS, who may respond to immunosuppression therapy, but at very low rates (2).

A number of underlying biological mechanisms, multiple causes of FSGS, and side effect profiles contribute to the present day challenge of identifying effective and acceptable treatments. Globally, research teams are seeking a better understanding of the underlying biological mechanisms of subgroups of patients with FSGS that may provide targets for future therapy (3). This paper will provide a summary of commonly used therapies for FSGS and present strategies for successful clinical trial design to support the testing of novel agents.

## Current Therapies for FSGS

A majority of therapies for FSGS (Table 1) have been either tested in phase 2 trials, but not used for registration (product labeling) or utilized without significant evidence as a treatment of last resort (3–7). The published estimates of efficacy vary widely from 25 to 69% across agents. Recent evidence supports that certain patient populations have a lower likelihood of responding to therapy. For instance, individuals with high risk APOL1 genotypes, found in individuals of African ancestry, have a higher likelihood of unfavorable outcomes (continued proteinuria, ESKD) (8–11). Furthermore, efficacy may also be predicted by response to prior therapy, such as glucocorticoids. Differences in such patient characteristics have important implications in study interpretation and trial design.

**Table 1:** **Commonly used therapies for FSGS in 2015**.

	Proteinuria remission (%)	Cost (1)	Monitoring (2)
Corticosteroids	25–59	$	+
Calcineurin inhibitors			
Cyclosporine	46–69	$$$	++
Tacrolimus	^a^	$$$	++
Mycophenolate	33	$$	++
Cyclophosphamide	27–55^b^	$	+++
ACTH	29^a^	$$$$$	+
Rituximab	38^b^	$$$$	+

*(1) Cost comparison of these agents is based on a course of therapy, with monthly costs ranging from the approximately $30 ($) to $46,000 ($$$$$)*.

*(2) Monitoring frequency is labeled for frequency to screen for side effects, drug level, and therapeutic effects. Increasing number of + are used to show increasing frequency of standard lab monitoring*.

*^a^The expected response to calcineurin inhibitors is approximately the same. Publications that have compared the two show similar efficacy but a worse adverse event profile with cyclosporine*.

*^b^Results are reported from steroid resistant and steroid sensitive patients*.

The initial selection of an appropriate therapeutic regimen by the treating physician is related to the anticipated likelihood of disease control (proteinuria resolution, preservation of kidney function) and the safety profile of the therapeutic agent. In the absence of more precise biomarkers, the subsequent tailoring of therapeutic regimens for patients are driven by disease characteristics (treatment response), side effect profile, or cost/convenience factors. Table 2 summarizes the common side effects that may influence differential treatment selection.

**Table 2:** **Common side effects reported with treatment of FSGS**.

Medication	Common side effects
Corticosteroids	Weight gain, hyperglycemia, hypertension, osteopenia, mood changes, weakness
Calcineurin inhibitors^a^	
Cyclosporine	Hypertension, gingival hyperplasia, hypertrichosis, infection
Tacrolimus	Hypertension, infection, tremor
Mycophenolate	Nausea/diarrhea, leukopenia, teratogenic, infection
Cyclophosphamide	Nausea, leukopenia, infection, alopecia, teratogenic
ACTH	Weight gain, hypertension, rash, acne, hypertrichosis, mood changes, weakness
Rituximab	Infusion reaction, infection, leucopenia

*^a^Calcineurin inhibitors have an uncommon side effect of nephrotoxicity, which may influence clinicians and patients about initial use or duration of therapy*.

## Current Clinical Trial Designs and Statistical Considerations for Rare Diseases

Since the Orphan Drug Act was passed in 1983, an increased number of drugs and biologics have been approved for rare diseases in the US (12). An orphan drug is defined as one targeted toward rare diseases (prevalence <200,000 persons) or disease with greater prevalence but for which the cost of drug development is expected to not be recoverable from US sales (13). In Europe, rare diseases are defined as life-threatening or chronically debilitating conditions that affect ≤5 in 10,000 people in the EU (Official Journal of the European Union 2009/C 151/02).

Although there is an ethical imperative to hold clinical trials in rare diseases to rigorous ethical and scientific standards, analyses of rare vs. non-rare clinical trials indicate that there are differences in design characteristics. Trials in orphan drugs are more likely to be smaller, non-randomized, lack blinding, and use disease response instead of progression or survival endpoints (14–16).

## Changing the Paradigm

The utilization of traditional clinical trial designs may not be appropriate for rare diseases. Fortunately, a myriad of design and analysis options (see Table 3) exist in the clinical trials and statistical literature that allow for the study of drugs in rare diseases, such as FSGS, that meet stringent effectiveness and safety standards. A clinical trial development program of studies in FSGS should result in evidence that provides confidence for patients, regulators, investigators, and clinicians, given the current therapeutic options and knowledge base.

**Table 3:** **Brief glossary of clinical trials terms**.

Term	Brief description
3 + 3 trial design	A conventional and popular phase 1 dose escalation design that estimates the MTD^a^ by sequentially studying cohorts of size 3
Adaptive design	A clinical study design that uses accumulating data to decide how to modify aspects of the study as it continues, without undermining the validity and integrity of the trial (17)
Bayesian methods	Bayesian methods use prior information on the differences between treatments before the trial is completed, and update this information based on data obtained from the trial. The difference between treatments is not a single fixed parameter in the Bayesian approach; rather, a distribution of potential values characterizes treatment differences
Continual reassessment method (CRM)	CRM is an adaptive dose-finding study design that uses Bayesian methods to estimate the MTD. It frequently results in fewer adverse events and more accurately estimates the MTD
Crossover design	A clinical trial design in which participants receive a sequence of different treatments, resulting in within-subject comparisons that generally reduced the required sample size. This design is in contrast to the parallel-group design where participants receive only one protocol-specified treatment
Dose limiting toxicity (DLT)	Severe but (ideally) reversible adverse events that occur within a generally short protocol-defined period
Frequentist methods	A framework of statistical inference that is generally taught in most introductory statistical courses, that treats the difference between treatments as an unknown and fixed parameter. Clinical trial results are considered from the perspective of multiple independent repetitions of the experiment which sometimes cause difficulties in the interpretation of results
“Learn and confirm” clinical trial paradigm	An alternative to the traditional “phased” approach to drug development (i.e., phase 1, 2, and 3). The goal of the learning phase is to assess the relationship between the dose and administration of a new drug and its expected efficacy and safety. The goal of the confirming phase is to capitalize on the more complete information obtained in the learning phase to efficiently study the risk-benefit of the new agent
Maximum tolerated dose (MTD)	The highest dose of a drug or treatment that does not cause unacceptable side effects (18)
N-of-1 design	Single-subject clinical trial that has the goal of determining the best intervention for an individual patient based on objective criteria
Seamless trial designs	Clinical trial designs that address, within a single trial, objectives that are normally achieved through separate trials

*MTD, maximum tolerated dose*.

Classical drug development employs a “learn and confirm” paradigm over a series of steps. The drug development pipeline begins with discovery with preclinical *in vitro* and *in vivo* animal experiments and toxicology studies. Phase 1, phase 2, and proof-of-concept studies in human participants follow in the exploratory phase, with (generally) two adequate and well-controlled phase 3 studies conducted in the full development confirmatory phase. After approval, post-marketing phase 4 studies are often conducted to provide additional characterization of the efficacy and safety in a broader patient population. Generally, each step involves a separate study during the human studies stage.

**Figure 1:**
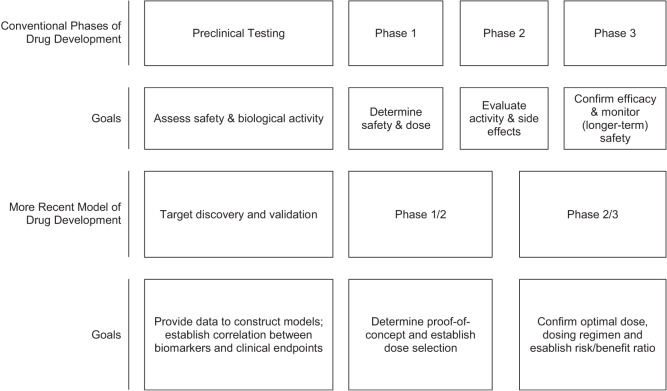
**Models of drug development**.

This classical drug development process is often not feasible in rare diseases. In this setting multistage designs, particularly adaptive designs, and seamless phase 1/2 or phase 2/3 trials may be used to maximize information while minimizing the strain on the available patient population [based on Orloff et al. (61)] (Figure 1). Adaptive designs refer to studies that include “a prospectively planned opportunity to modify one or more specified aspects of the study design and hypotheses based on analysis of study data (usually interim data)” (19). Adaptive trials have been a recent topic in the clinical trials literature, with seminal papers in 2006 (17, 20–22); however, the concept has been around since the 1970s in medical trials with adaptive randomization and sequential designs (23). In addition, Bayesian methods offer an alternative statistical approach to inference (relative to the frequentist or classical approach) that treats probability as a measure of the degree of personal belief instead of the frequency achieved in long-run repetitions of an experiment. Bayesian methods have gained acceptance since the early 2000s, with use of the phase 1 continual reassessment method (CRM; Bayesian in everything but name) (24), and Bayesian stopping rules in phase 2 trials (25). The literature on trial design in rare diseases or small sample size situations is redolent with suggestions for use of Bayesian methods (26–29).

These novel designs have been successfully used in a variety of therapeutic areas, including rare and common diseases. A modification of the time-to-event CRM approach (TITE-CRM) was used in a phase 1 study of continuous MKC-1 in patients with advanced or metastatic solid malignancies (30). The study identified a maximum tolerated dose by studying 24 patients, and concluded that the adaptive Bayesian design allowed for a more efficient dose escalation and also allowed for late toxicities. Kaufmann et al. (31) designed a multicenter adaptive two-stage phase 2 trial of CoQ10 in amyotrophic lateral sclerosis (ALS) to identify an appropriate dose in stage 1 and then compare the selected dose against placebo for futility in stage 2. They concluded their adaptive design avoided the need for a much larger conventional phase 3 trial. Finally, an adaptive seamless phase 2/3 randomized trial of dulaglutide combined with metformin in type 2 diabetes patients efficiently (32) explored a large number of doses and selected two doses for head-to-head comparison with sitagliptin; both dulaglutide doses demonstrated superior glycemic control (33).

### Study Population

A common approach to the testing of novel agents for FSGS or other conditions will select a patient sample that has demonstrated resistance to standard therapies, positioning the novel agent as a salvage therapy. Depending on the investigational drug target, this approach may doom the agent to failure as the patients on study may have already entered a late or irreversible phase of the disease. FSGS targeted therapies may be best suited to patients in early or mid-disease where the potential for the drug to demonstrate activity against a molecular target can be shown. Enrichment designs represent a unique opportunity in FSGS by selecting patients who are more likely to respond to therapy, based on specific biomarkers. Such designs reduce sample size by reducing patient heterogeneity, improving the chance of successful enrollment. The development of biomarkers and targeted agents targeted agents in earlier phases of drug development in FSGS should help in assessing whether this strategy would be advantageous in later confirmatory stages (34).

Finally, FSGS affects patients of all ages. As the implications of uncontrolled FSGS resulting in kidney failure are similar across the lifespan, drug development strategies should include children in every setting where drug safety has not shown specific additional risk in immature preclinical testing.

### Endpoints

The goal of FSGS therapies is the normalization of urinary protein excretion [measured urine protein/creatinine ratio (UP/C)], preservation of kidney function (3), restoration of the patient health, and avoidance of adverse events (3). Efficacy endpoints currently being used in many FSGS trials include the proportion of participants who achieve complete remission (UP/C <0.3 g/g and preserved kidney function) and partial remission (50% UP/C reduction and <3 g/g and preserved kidney function). These endpoints have been justified based on two retrospective observational studies demonstrating the relationship between remission status and long-term renal survival (35, 36). The timing for achieving remission is generally assessed at 3–6 months after initiation of study medication. Characterizing response as a binary outcome generally leads to larger sample sizes (37). Smaller clinical trials may be achievable in the confirmatory stages of development in FSGS, for similar Type I and Type II error rates, if UP/C is analyzed as a continuous outcome. For example, multiple measures of UP/C can be collected with analyses based on longitudinal methods [see, for example, the approach of Greene et al. (38) for eGFR endpoints]. Research is needed to better understand the differences in UP/C that are clinically meaningful and would result in changes in clinical practice.

### Learning and Confirmatory Phase Goals and General Considerations

During the learning phase of drug development in FSGS, we investigate the correct dose range by investigating preliminary efficacy and ensuring that the drug meets minimal requirements for dose-limiting toxicity and tolerance. In the confirmatory phase, the goal is to obtain sufficient efficacy and safety information in well-controlled trials to support its acceptance. The control group may be placebo (superiority trials) or an active control (superiority, non-inferiority, or equivalence trials). The highest levels of medical evidence are achieved through the use of randomization, blinding, and concurrent controls, and there should be strong rationale for *not* using these features in FSGS trials.

Crossover designs and N-of-1 trials have been suggested for rare diseases (27) because they reduce sample size by allowing for within-subject treatment comparisons. In FSGS confirmatory trials, where the endpoints may be measured at 6 months or later, there are design implications of period effects where the disease is not stable over the time course of the two treatment periods.

The impact of design and analysis decisions should be fully evaluated. Frequentists calculate sample size based on the hypothesis testing framework that specifies the type I error, power, and expected treatment difference. The Bayesian approach does not employ a strict need to calculate sample size because the goal is to update prior beliefs about the null hypothesis with the data. In past FSGS trials, the traditional, equal-allocation, fixed sample-size design was most commonly used. Clinical trials simulations can be used for future trials to assess the tradeoffs between frequentist and Bayesian options, assessing characteristics of the design for specific agents and phases, such as probability of stopping for futility incorrectly and sample size options to achieve a certain decision criterion.

### Adaptive Designs

An adaptive design is defined as “a clinical study design that uses accumulating data to decide how to modify aspects of the study as it continues, without undermining the validity and integrity of the trial” (17). An adaptive design requires access to accumulating data at multiple stages of the trial (20). Adaptation rules applied at each interim analysis may affect: how a subject will be allocated to the available arms of the trial, how many subjects will be sampled at the next stage, when to stop the trial for efficacy, harm or futility, and other decision features of the trial (20). An important prerequisite for an adaptive trial is that the accumulating information on the end point can be assessed quickly enough to trigger the adaptive decisions relative to the enrollment rate. FSGS trials typically meet this criterion because the prevalence of the disease results in slow enrollment and normalization of urine protein excretion can be observed as early as 2–3 months depending on the agent under investigation.

In early phases of FSGS development, the following adaptive approaches may be most useful:

○ *Adaptive dose finding* is used in early phase clinical development to identify the minimum effective dose and/or the maximum tolerable dose, which is used to determine the dose level for the next phase clinical trials. In particular, the continual re-assessment method allows assessment of dose-limiting toxicity in small cohorts at a given dose (one to four patients commonly), updating the dose-toxicity curve, and treating additional cohorts until a pre-specified level of certainty is achieved (39).
○ *Drop-the-loser or play-the-winner design* allows for dropping an inferior treatment group or maintaining a superior treatment group. A drop-the-losers design also allows adding additional arms. These designs would be useful in FSGS when there are uncertainties regarding the dose levels for a new agent. These two approaches can be considered types of adaptive randomization methods, where the probability of being assigned to a dropped arm is 0 (see below).
○ *Adaptive seamless 1/2 trial designs* address within a single trial objectives that are normally achieved through separate trials in phase 1 and phase 2 of clinical development.

Adaptive designs that may be more useful during later stage drug development in FSGS include

○ *Sequential designs* allow for prematurely stopping a trial due to safety, futility, or efficacy (benefit) with options of additional adaptations based on results of interim analysis. Types of sequential designs include group sequential designs that employ repeated significance testing at pre-specified interim analysis times and boundaries approach where the amount of information and treatment effect size are assessed multiple times during the study (40–42). With these designs, the final sample size is unknown at the trial initiation, but sample sizes are generally smaller than a classical fixed sample size approach (40).
○ *Sample size re-estimation design* allows for sample size adjustment based on interim analyses. When limited information is available *a priori* on the variance of the outcome (e.g., variance of change from baseline to month 3 in UP/C) or the estimated treatment effect (e.g., proportion of participants who achieve complete remission), sample size re-estimation is attractive. A common method is to calculate the conditional power that a treatment difference will be observed at the end of the trial, given the current information. Hybrid methods are often used where the trial may continue with the original sample size if the conditional power if sufficient, may be stopped for futility if the increase is sample size is too great, or may be enlarged if neither of the other conditions is met (43).
○ *Adaptive randomization design* allows modification of randomization schedules based on varied or unequal probabilities of treatment. It is a type of allocation rule that determines how new patients are assigned to treatments dynamically (20). One such type of adaptive randomization is response-adaptive randomization where the allocation probabilities are unbalanced to provide a greater likelihood that treatments having more favorable outcomes are assigned.
○ *Adaptive seamless 2/3 trial design* addresses within single trial objectives that are normally achieved through separate trials in phase 2b and phase 3 of clinical development.

### Bayesian Methods

The difference between treatments is assumed to be an unknown and fixed parameter in the frequentist framework, whereas the treatment difference is not a single fixed parameter in the Bayesian approach. It is characterized by a distribution of potential values (26, 44). Bayesian methods use prior information on the differences between treatments before the trial is completed, and update this information based on data obtained from the trial producing a posterior distribution that can then subsequently be used as the prior for the next interim analysis or stage of a trial. This approach is attractive in the context of seamless phase 1/2 and phase 2/3 trials where smooth transitions between stages can occur as we learn more about treatment differences as the data accumulate (45, 46). However, there are many examples of adaptive designs that use both Bayesian (47) and frequentist approaches (47–49).

A key stumbling block in Bayesian methods is elicitation of the prior distribution of the treatment difference and the degree of certainty and subjectivity (27). Information on the prior distribution for the unknown treatment effect can be determined from data from the literature (e.g., a single drug approved for another indication, characteristics for a class of similar agents) or from expert knowledge (28). The biases that may result from expert opinion have been widely discussed and heuristics have been developed to minimize these biases (50–52). Hampson et al. (28) provide an example of elicitation of priors for a clinical trial in very rare diseases. Interestingly, the eliciting of prior beliefs may provide benefits for frequentist clinical trials in considering the magnitude of effect for determining sample size and assessing the level of evidence needed to convince the clinical community to change practice (53).

Both the use of adaptive design methods and the Bayesian approach in clinical trials is consistent with the FDA’s *Clinical Path Initiative* (54) that was originally developed to deal with the problem of increased trial spending without a resulting increase in the success rate of new drug approvals. The FDA advocated for advancing innovative trial designs, capitalizing on use of prior experience, or accumulating information in a trial. Dr. Woodcock, then Acting Deputy Commissioner for Operations at the FDA, emphasized two points: (1) there should be scientific evidence that a drug or biological works and (2) there should be a degree of certainty about the prediction that the new product works. She notes that the law does not tell us exactly what the degree of certainty should be, but a level of evidence from the current development that depends somewhat upon the prior knowledge base – whether mechanistic or more general. Thus, the extension of these ideas from common diseases to rarer diseases, such as FSGS, seems a natural step in clinical trials methodology. As with conventional clinical trials designs, the new proposed paradigms do not compromise the ethical imperative to protect human subjects according to such guidelines as the Belmont Report and the Declaration of Helsinki.

## Example

One context where using adaptive and Bayesian methods in clinical trials can benefit advances in FSGS therapies is in dose-finding trials. The methods that are used for dose-finding trials in non-rare diseases could be costly and imprecise. We can reduce the sample size needed for estimating the maximum tolerated dose while maintaining precision by using the CRM first presented by O’Quigley et al. (55). We compare the most common method for dose-finding clinical trials (the 3 + 3 design) to the CRM using simulations run under reasonable settings for FSGS.

The two main goals of dose-finding clinical trials are to put as many subjects as possible in the dose closest to the maximum tolerated dose (MTD) and to estimate the MTD as accurately as possible. The MTD is defined by the National Cancer Institute as “the highest dose of a drug or treatment that does not cause unacceptable side effects” (18). Unacceptable side effects are determined by the proportion of subjects in the population who would have a dose limiting toxicity (DLT). In our FSGS example, we consider a >30% decrease in eGFR to identify a DLT and 30% of the study population having DLTs at a dose defines the MTD. Generally, to obtain the best estimates of the dose–response relationship, we would want to allocate patients equally to all doses to gain as much information as possible. However, this is contrary to the goal of treating as many patients with the currently estimated MTD. Therefore, we compare the 3 + 3 and CRM methods on how often each selects the correct MTD dose and how many subjects are allocated to the MTD dose to evaluate the ability of these methods to meet both goals simultaneously.

### Methods

As described by Storer (56), the 3 + 3 method assigns the first three subjects in the lowest dose group. If no subjects have a DLT, then the next three subjects receive the next highest dose. If one subject out of the original three has a DLT, then the next three subjects are allocated to the same dose. If two or more subjects have DLTs, then the trial is stopped and the MTD is selected to be the next lowest dose. If the first dose has more than one DLT, then it is selected as the MTD. Because many drugs assessed in FSGS trials have been developed for other indications, we limit the number of doses studied to four in this example; thus, the maximum sample size is 24 with the 3 + 3 design.

The CRM is an adaptive and Bayesian method for dose-finding trials in which we use prior information combined with collected data to help find the MTD (Bayesian) and give future subjects doses based on estimates from collected and prior information (adaptive). After each subject is enrolled and treated, the outcome information is combined with prior information and previous outcomes to update the dose–response curve from which the MTD is estimated and given to the next subject. The sample size in CRM can be fixed or variable. To provide a fairer comparison with the 3 + 3 design, we used an early stopping rule to select the estimated MTD as the dose which is assigned seven times in the trial (which limits the maximum sample size to 24). Stopping rules of this kind were first used by Korn et al. (57) and further discussed by O’Quigley (58).

For both 3 + 3 and CRM designs, we ran 10,000 trial simulations for four different true DLT probability scenarios. For each scenario, we compared the proportions of dose selected, the proportion of doses assigned, and the average and SD of sample sizes for both designs. We chose to always include the MTD of interest as one of the doses in our simulations for ease of comparison of trial types. The simulations were run using R and the package dfcrm created by Cheung (59, 60).

### Results

In Scenario 1, the CRM selects the correct dose about 62% of time where the 3 + 3 design identifies the MTD just under 35% of the time (Table 4). The 3 + 3 design also chooses dose 1 in this scenario 50% of time, meaning that half of the time we would expect the 3 + 3 to identify the estimated MTD to be a dose where no DLTs would happen. There are two doses in Scenario 2 in which the true DLT proportion is 30%, which are identified by the CRM 72% of the time and by the 3 + 3 design <50% of the time. Scenario 3 and Scenario 4 show the CRM is doing a better job at selecting the correct dose by approximately 10% over the 3 + 3 design. In Scenario 4, the 3 + 3 design again selects a dose as the estimated MTD where no DLTs would occur nearly 40% of the time.

**Table 4:** **Percentage of time true DLT dose is selected by design in 10,000 simulated trials**.

Dose scenario	Design	Dose			
		1	2	3	4
Scenario 1: (0.00, 0.30, 0.40, 0.60)	3 + 3	50.3	**34.6**	14.6	0.6
	CRM	10.9	**62.2**	24.1	2.8
Scenario 2: (0.05, 0.05, 0.30, 0.30)	3 + 3	5.0	48.6	**26.5**	**19.9**
	CRM	6.1	21.5	**46.7**	**25.7**
Scenario 3: (0.05, 0.10, 0.15, 0.30)	3 + 3	11.7	16.5	41.3	**30.5**
	CRM	7.1	12.9	37.6	**42.4**
Scenario 4: (0.00, 0.25, 0.30, 0.45)	3 + 3	39.7	30.6	**25.0**	4.6
	CRM	5.9	47.8	**33.9**	12.4

*The bolded value is the true DLT dose*.

The CRM tends to have about 12 subjects needed for the trial with SDs around 2 for all scenarios investigated (Table 5). On the other hand, the 3 + 3 design shows greater fluctuations in mean sample size (10.1–14.4) with larger SDs (approximately 3.5 for each simulation) than the CRM designs.

**Table 5:** **Mean (SD) sample size by design in 10,000 simulated trials**.

Dose scenario	Design	
	3 + 3	CRM
Scenario 1: (0.00, 0.30, 0.40, 0.60)	10.1 (3.2)	12.0 (1.7)
Scenario 2: (0.05, 0.05, 0.30, 0.30)	13.3 (3.5)	11.8 (2.0)
Scenario 3: (0.05, 0.10, 0.15, 0.30)	14.4 (3.7)	11.9 (2.2)
Scenario 4: (0.00, 0.25, 0.30, 0.45)	11.3 (3.7)	12.1 (1.7)

Our small simulation study shows the advantages of a Bayesian adaptive approach (CRM) over a more traditional clinical trial dose-finding design (3 + 3) in terms of assigning as many patients to the MTD as possible and selecting the correct MTD. In the long term, using the CRM over the 3 + 3 design could lead to enormous financial and time savings by increasing the probability that we move to further phases of clinical trials with the correct dose. Although there is no clear winner between the trial types for sample size under all scenarios investigated (Table 5), the stability of the average sample size and smaller SD when using the CRM would allow investigators to be more confident in expected sample size needed in the planning stages of the trial. Using the CRM may also decrease the concern of running a trial which uses a sample size closer to the maximum number of 24 subjects.

## Conclusion

Focal segmental glomerulosclerosis therapies are challenging based on incomplete efficacy and safety information, leading to the inability to define the right agent for the right patient. Novel agents that are based on molecular profiling are emerging which will benefit from an enriched trial eligibility approach. While enrichment may improve signal, trials will need to be designed for feasibility in FSGS endophenotypes defined by molecular profiling and target-relevant biomarkers. Rational application of more modern clinical trials designs, that have found increasing acceptance in the pharmaceutical, regulatory, and academic environments, increases the chance of successful studies that evidence of safe and effective therapies in rare diseases, such as FSGS.

## Author Contributions

All authors give final approval to publish this work and agree to be accountable for all aspects of the work in ensuring that questions related to the accuracy or integrity of any part of the work are appropriately investigated and resolved. CS, JJ, DS, SM, JT, and DG: conception and design of the work, drafting the work, and revising it critically for important intellectual content.

## Conflict of Interest Statement

DG serves as a consultant under contract between University of Michigan and the following companies: GlaxoSmithKline, Bristol-Myers Squibb, Janssen, and Retrophin. The remaining authors declare that the research was conducted in the absence of any commercial or financial relationships that could be construed as a potential conflict of interest.
